# Staged Correction of Severe Recurrent Clubfoot Deformity With Dislocation of the Chopart Joint Using a Hexapod External Fixator and Unconventional Arthrodesis

**DOI:** 10.5435/JAAOSGlobal-D-21-00116

**Published:** 2022-04-07

**Authors:** Allen Kadado, Noel Osereimen Akioyamen, Rachel Garfinkel, Nickolas Nahm, Ferras Zeni

**Affiliations:** From the Department of Orthopaedic Surgery, Henry Ford Hospital, Detroit, MI.

## Abstract

Despite success of the Ponseti method, a subset of patients with clubfeet experience residual deformity. Surgical release after unsuccessful serial casting can lead to residual clubfoot deformities, including a flat-top talus. We present a case of a 17-year-old boy with a dysmorphic ankle and a complete dorsal dislocation of the Chopart joint. Because of pain with activities and functional limitations, the patient underwent a staged correction of the dislocation. The deformity was corrected through a staged approach using a Taylor Spatial Frame, navicular excision, talocuneiform arthrodesis, and calcaneocuboid arthrodesis. One year postoperatively, the patient is pain free with notable functional gains.

Talipes equinovarus, also known as clubfoot, is a congenital deformity of the lower limb marked by varus and equinus in the hindfoot, cavus in the midfoot, and adduction in the forefoot.^1^ Clubfoot has a prevalence between 1 and 2 per 1,000 births, making it one of the most common musculoskeletal birth defects.^2^ The exact pathogenesis of clubfoot has yet to be discovered, although it is generally thought to have polygenic and environmental components.^3,4,5,6^ An untreated clubfoot causes pain and disability with a potential to reduce quality of life and increase morbidity and mortality.^7^ Thus, early diagnosis and intervention of clubfoot is imperative. Available treatments for clubfoot include the Ponseti method, French method, soft-tissue procedures, osteotomies, and use of external fixators in the most resistant cases.^8,9^ Although the current trend in contemporary clinical practice is to correct severe clubfoot deformity using external fixation, Radler and Mindler^10^ demonstrated the importance of a case-by-case analysis for unique pathoanatomy in successfully treating clubfoot deformity.

In this case, bilateral clubfoot was initially treated with the Ponseti method. This method is the standard treatment of congenital clubfoot with excellent outcomes, although it does have a potential for recurrence due in part to patient noncompliance.^11,12^ In our patient, Chopart joint dislocation was noted at the time of his presentation in adolescence. This may have been related to excessive release or overcorrection of the clubfoot, although this conclusion was not entirely corroborated because of unavailability of surgical records and inability of the family to provide sufficient details regarding surgical treatment. Correction of these deformities required an unconventional approach, including staged surgical intervention, joint distraction, bone resection, and joint fusion.

The patient was informed that data concerning this case would be submitted for publication, and he provided consent.

## Case Report

A 17-year-old boy presented for the evaluation of bilateral foot pain, left worse than right. The patient's history included bilateral congenital talipes equinovarus diagnosed at birth, which was treated using the Ponseti method with serial casting followed by Achilles lengthening. He subsequently had tibialis anterior tendon transfer at the age of 5 years. He potentially also had soft-tissue releases at this time; however, this is unclear. The patient described generalized foot and ankle pain for the majority of his adolescence. The pain gradually worsened as he became older and more involved in athletic activities, ultimately limiting his function and participation. There was no notable injury, no difference in pain when barefoot or in shoes, and no consistent pain medication requirement.

**Figure 1:**
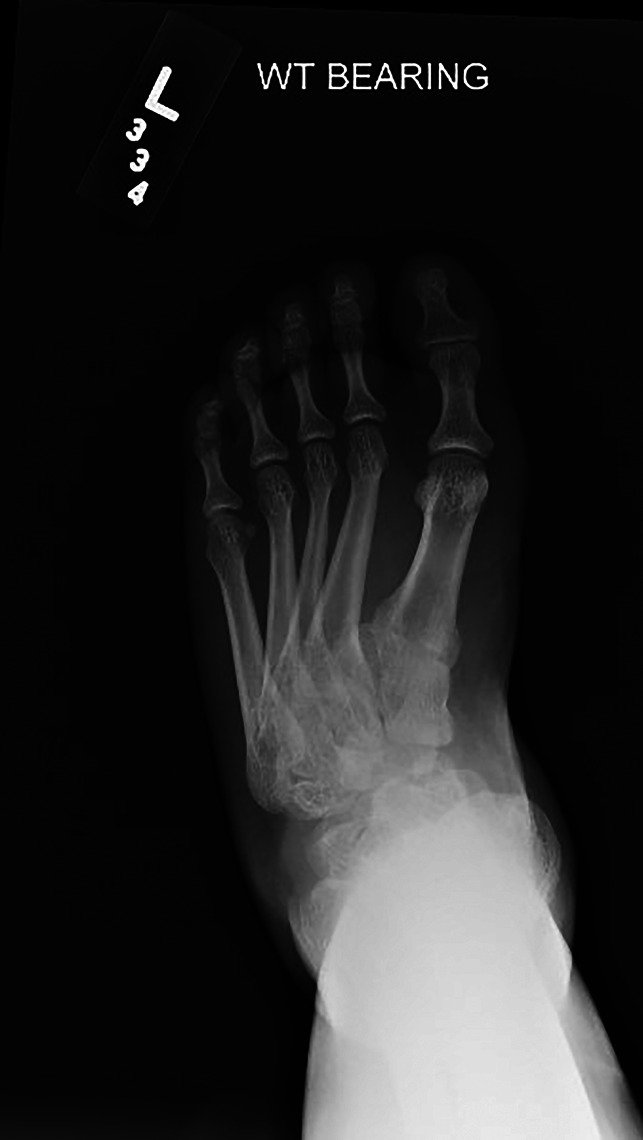
Anteroposterior preoperative radiograph of the left foot.

**Figure 2:**
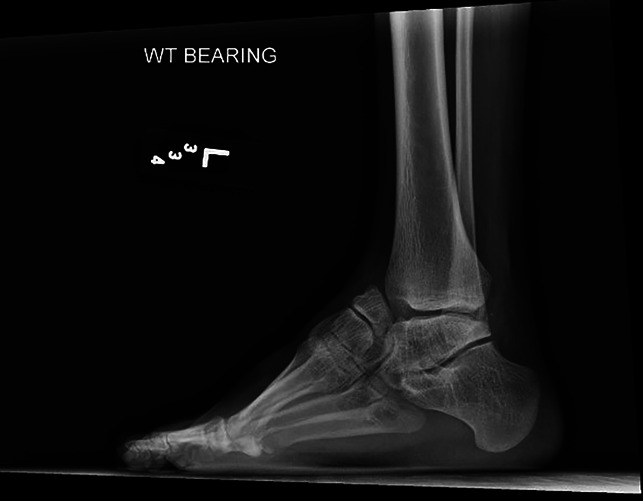
Lateral preoperative radiograph of the left foot and ankle.

**Figure 3:**
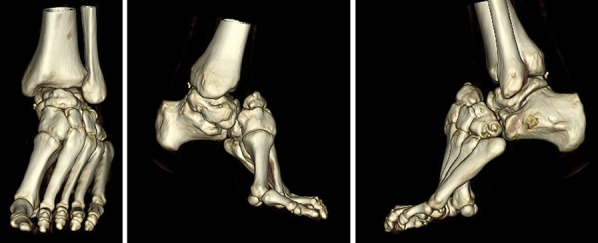
Three-dimensional CT representations of the left foot and ankle demonstrating notable equinovarus deformity with chronic dislocation of the Chopart joint and resultant talus and navicular dysplasia.

Physical examination of the left lower extremity demonstrated shortening of the left foot with bony prominence dorsally. Medial and lateral incisions were noted on the foot from prior surgery. Ankle dorsiflexion was neutral, plantar flexion was 20°, and hindfoot inversion/eversion was limited. The patient demonstrated 5/5 strength ankle plantar flexion, 4/5 strength ankle dorsiflexion, and limited eversion strength. No evidence of neurovascular compromise was observed. Examination of the right lower extremity was essentially the same, except for lesser deformity and slightly improved range of motion. The patient stood with notable hindfoot varus. Weight-bearing radiographs of the bilateral foot and ankle demonstrated multiple significant deformities on the left (Figures 1 and 2). Notably, there was complete dorsal dislocation of the talonavicular joint with the first and second ray dorsiflexed with respect to the hindfoot. Complete flattening of the talar dome, a dysmorphic ankle joint, and early tibiotalar osteoarthrosis were also observed. Similar findings were seen on the radiographs of the right side, with the talonavicular joint subluxated rather than dislocated. Both feet showed evidence of notable cavus deformity. CT scan and three-dimensional reconstruction images redemonstrated dorsal dislocation of the Chopart joint (Figure 3).

**Figure 4:**
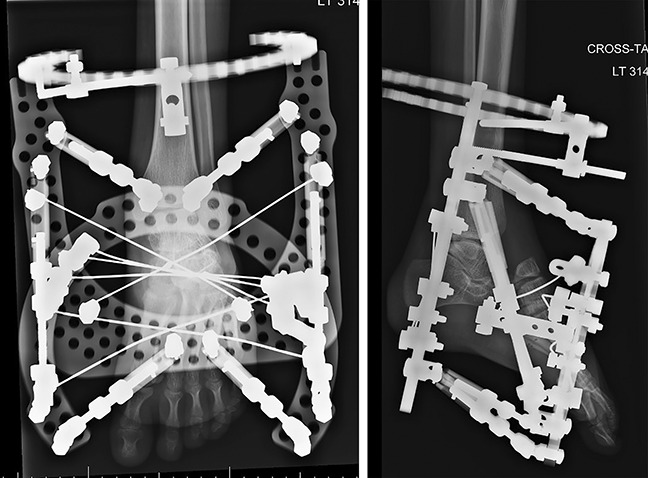
Anteroposterior and lateral postoperative radiographs (early stage 1) demonstrating the application of the hexapod fixator.

**Figure 5:**
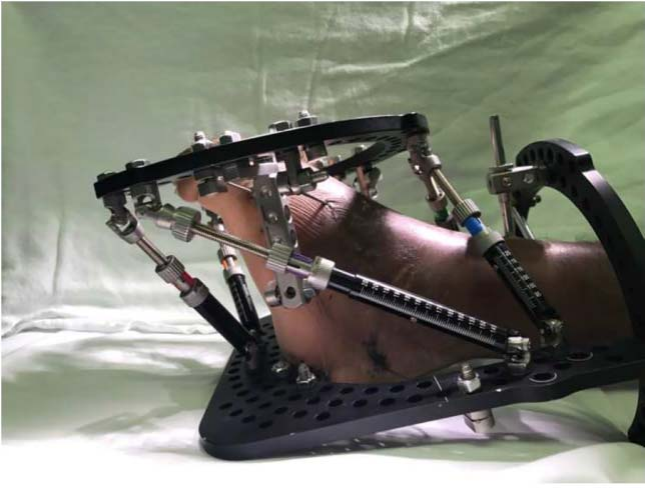
Clinical image demonstrating frame orientation.

A two-stage surgical intervention for the left foot was agreed upon, with the goal of correcting the deformity to improve pain and function. A hexapod external fixator (Taylor Spatial Frame [TSF]) was initially used to lengthen the foot and reduce the talonavicular joint before fusion. The frame was mounted with struts aligned in the longitudinal axis of the foot (Figures 4 and 5).

**Figure 6:**
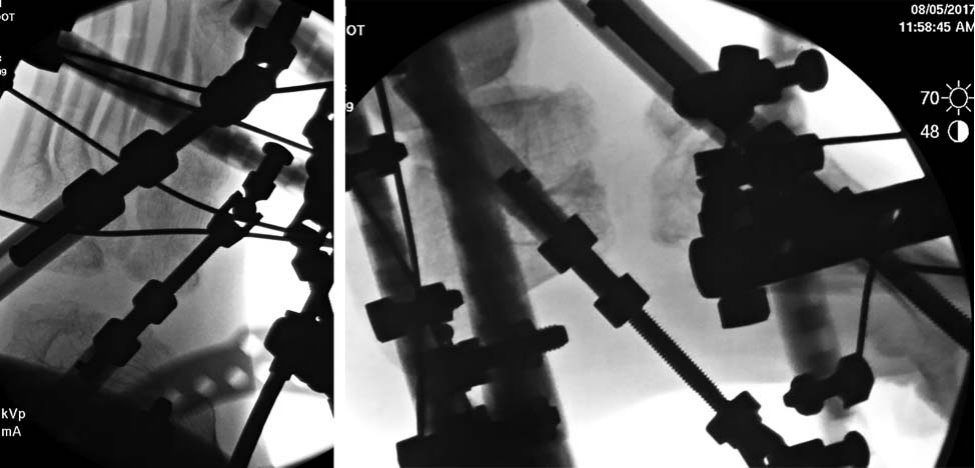
Anteroposterior and lateral radiographs (late stage 1) demonstrating gradual distraction through the Chopart joint using the hexapod fixator.

The patient's postoperative course after the first stage was normal, and he was discharged home with the frame on postoperative day 1. He was given education and instruction regarding his strut protocol and discharged on Norco every 4 hours as needed for 1 week. On postoperative day 7, the patient was doing well with no complications or pain. At this point, the struts of the TSF were adjusted to accommodate a new program. On postoperative day 14, the patient reported mild toe discomfort. Physical examination was normal. His radiographs demonstrated excellent distraction across the talonavicular and calcaneocuboid joints (Figure 6); however, reduction of the talonavicular joint was not noted at this time. Careful monitoring of the frame and associated struts was important to ensure that there was no wire displacement and that the correction progressed as planned.

**Figure 7:**
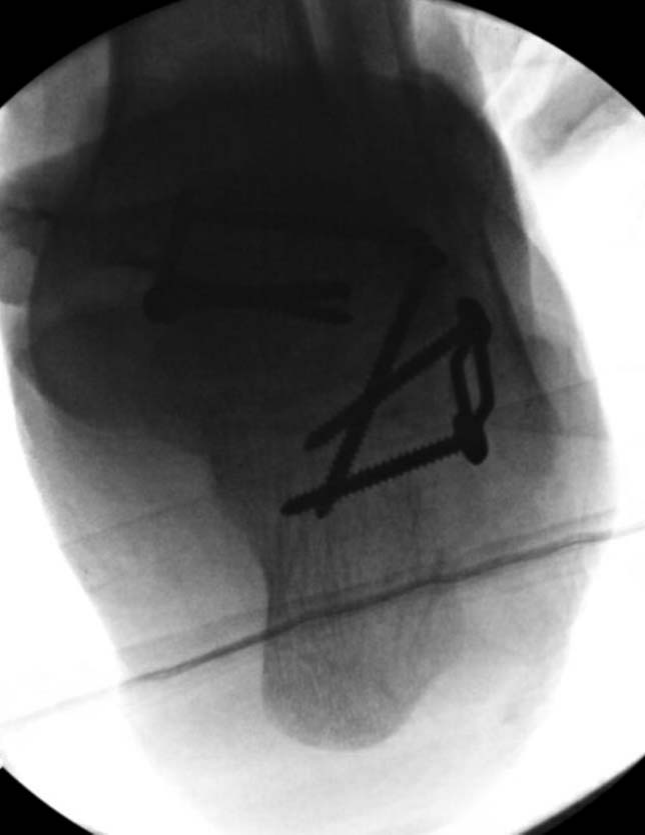
Hindfoot radiograph of the foot intraoperatively after the hexapod frame was removed and arthrodesis was completed.

The initial planned interval between stage 1 and stage 2 was 2 weeks; however, there was notable delay because of issues with insurance authorization. On postoperative day 31, the patient returned for the second stage of the procedure involving removal of the TSF followed by arthrodesis. Although our goal was to use the TSF to allow for anatomic midfoot fusion, intraoperatively, the decision was made to excise the hypoplastic navicular joint because of the severity of its shortening and its dysmorphic osteology. Navicular retention would have posed a difficult surgical challenge in terms of fusion to the talus, so it was instead used as a bone graft for subsequent fusion, and the TSF acted as an instrument to prepare misfit fusions. A balanced resection of the calcaneocuboid joint was done to match the degree of shortening medially. In preparation for the talocuneiform arthrodesis, approximately 2 to 3 mm of the talar head was resected to create a flat and broad surface area for fusion. Lag screws were placed across both articulations followed by compression plating medially and laterally. The final radiograph of the patient's foot showed that he was in slight hindfoot varus (Figure 7). He was placed in a short leg splint, made non–weight bearing, and discharged home on postoperative day 1 with Norco every 4 hours as needed for 1 week.

The postoperative course was normal. On postoperative day 23, the patient returned to the clinic for suture removal and recasting. Non–weight bearing was maintained for 2 months. Radiographs at the 2-month mark demonstrated fusion. He was transitioned to a boot, started physical therapy, and progressed to full weight bearing and told to wean himself out of the boot. At 3 months, fusion progressed and was satisfactory. Subsequently, the patient was allowed to engage in unrestricted activities, including sporting activities. Ankle range of motion was 5° of dorsiflexion with 10° to 15° of plantar flexion. The alignment of the foot was noted to be neutral. The patient reported use of regular shoes and increased activity level. No pain was observed on palpation of the medial or lateral hindfoot and the anterior aspect of the ankle joint.

**Figure 8:**
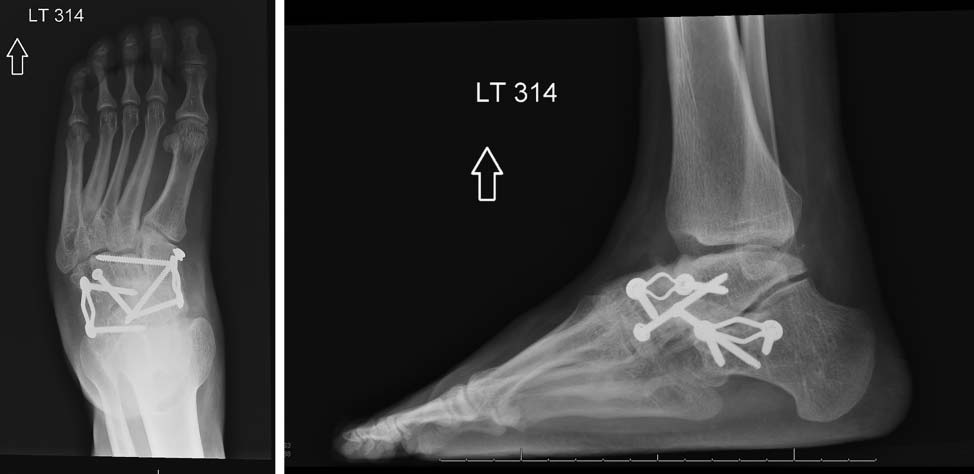
Anteroposterior and lateral radiographs 1 year after navicular excision, talocuneiform arthrodesis, and calcaneocuboid arthrodesis.

One year postoperatively, he denies foot and ankle pain. Radiographs demonstrated excellent talocuneiform and calcaneocuboid arthrodeses without any evidence of failure (Figure 8). The patient wears regular shoes, requires no bracing, and continues to be active. He reports excellent scores on multiple outcome assessments, including RAND Short Form-36, Patient-Reported Outcomes Measurement Information System Global Health, and American Orthopaedic Foot and Ankle Society Ankle-Hindfoot Scale.

## Discussion

Our patient presented with severe residual clubfoot deformity. The debilitating nature of the disease prompted surgical intervention. In the literature, no single standard treatment algorithm has been adopted; thus, the unique anatomy of each case should drive treatment. In exploring different options for our patient, we aimed to decrease pain and increase function, given his deformity and dislocation. Our approach avoided surgical soft-tissue releases and tendon transfers and instead used the TSF for distraction histiogenesis.^13^ Several existing studies report success in using the TSF to correct relapsed clubfoot.^14,15,16,17,18^ We used the TSF, which has been shown to provide precise and robust correction.^19,20^ In addition, we chose a two-stage correction because fewer complications have been reported when treating severe foot deformities with gradual correction.^21,22^

Current literature supports using adjunctive osteotomies for the correction of recurrent clubfoot deformity in older children.^23,24^ In our case, weighing risks and benefits, we performed a navicular excision to avoid potential complications. Arthrodesis is generally avoided in young patients because of concerns regarding motion and accelerated adjacent degeneration. Given the dysplastic nature of the dislocated Chopart joint, fusion was deemed the best method to maintain the correction achieved by the TSF; thus, arthrodesis serves as a powerful and durable option in certain cases.

Current literature also supports long-term results of patients who undergo midfoot arthrodesis. In the study by Swaroop et al, 12 of 13 patients who had midfoot arthrodesis in their adolescent years were symptom free 3 years out from surgery. Only one patient developed a nonunion that required additional surgery.^25^

## Conclusion

This is a case of severe recurrent clubfoot deformity that posed a unique anatomic challenge. Surgical treatment using a hexapod external fixator and unconventional arthrodesis as part of a staged correction demonstrates excellent outcomes at 1-year follow-up.
